# Trends in Mendelian randomization in neurological disease research: a bibliometric analysis

**DOI:** 10.3389/fneur.2025.1525481

**Published:** 2025-07-17

**Authors:** Shengnan Yin, Kudelati Zakeer, Zekun Ma, Miaomiao Zhao, Aierpati Maimaiti, Zengliang Wang

**Affiliations:** ^1^Department of Neurosurgery, The First Affiliated Hospital of Xinjiang Medical University, Urumqi, China; ^2^College of Health Management, Xinjiang Medical University, Urumqi, China

**Keywords:** neurological disease, Mendelian randomization, bibliometric analysis, review, research trends

## Abstract

Bibliometric analysis (BA) was used in this study to examine the current state and trends of Mendelian randomization (MR) in neurological disease research. The Web of Science database was searched between 1 January 2014 and 1 September 2024 to retrieve relevant literature. The volume of publications, research themes, collaborative networks, and geographical distribution were studied quantitatively. A keyword co-occurrence analysis identified prominent research hotspots, including stroke, cardiovascular disease, and genome-wide association studies. Furthermore, highly cited literature underscored the potential of MR to elucidate causal relationships between genetic variants and health outcomes. International collaborative networks indicate that China, the United Kingdom, and the United States are the most engaged in collaborative efforts within this domain. The findings suggest that MR methods hold significant potential for applications in the investigation of neurological disorders, highlighting the necessity of international collaboration to foster scientific advancement. Future research should prioritize enhancing interdisciplinary collaboration and conducting comprehensive explorations of disease mechanisms to aid in prevention and treatment.

## 1 Introduction

Neurological disorders constitute a broad category of conditions that affect the nervous system, which encompasses the brain, spinal cord, and nerves. According to the World International Health Organization classification, neurological disorders are divided into epilepsy, Alzheimer disease and other dementias, cerebrovascular diseases including stroke, migraine and other headache disorders, multiple sclerosis, Parkinson’s disease, neuroinfectious, brain tumors, traumatic disorders of the nervous system due to head trauma, and neurological disorders as a result of malnutrition. They exhibit considerable diversity, encompassing neurodevelopmental disorders that may manifest in early childhood, neurodegenerative diseases that typically emerge later in life, and recently emerging conditions such as those caused by the SARS-CoV-2 virus, which can lead to neurological complications (1). The objective of research in neurological disorders field is to gain insight into the fundamental mechanisms underlying. This knowledge can inform the development of more effective treatments and preventive strategies. The study emphasized the significance of elucidating the genetic basis of neurological disorders, which can facilitate the comprehension of disease processes and inform the design of precision therapies (2). Furthermore, the field of neurological research is of paramount importance in addressing health disparities and improving global brain health. For example, The World Health Organization’s Intersectoral Global Action Plan (IGAP) on Epilepsy and Other Neurological Disorders 2022–2031 delineates strategic objectives to mitigate the global burden of neurological conditions, underscoring the significance of brain health throughout the lifespan (3). Neurological disorders rank prominently as leading causes of disability and mortality on a global scale, with their inherent complexity and heterogeneity presenting substantial challenges to researchers (4). The complexity of neurological disorders requires researchers to adopt more precise and innovative approaches to explore their aetiology and therapeutic strategies (5).

MR has emerged as a powerful tool in neurological research, offering a means of inferring causal relationships between exposures and outcomes by leveraging genetic variants as instrumental variables (6). The advent of advanced genetic and epidemiological methodologies has highlighted MR as a promising innovative approach for causal inference, facilitating the exploration of the aetiology of neurological diseases and the identification of potential therapeutic targets (7). This method is founded upon the tenets of Mendel’s laws of inheritance and instrumental variable estimation techniques, which permit the deduction of causal effects in the context of unobserved confounding (8). The details of it involve genetic instrument selection, assumption of validity, data sources, statistical analysis, sensitivity analyses and result interpretation. Systematic evaluation using MR analysis has identified multifactorial causal associations for Alzheimer’s disease, including novel therapeutic targets such as CD33, TBCA, VPS29, GNAI3 and PSME1 (9). MR uses genetic variants as instrumental variables to establish causality, which is particularly useful in neurological research where traditional randomize-controlled trials are often not feasible. This method has been instrumental in elucidating the aetiology of several neurological diseases, including Alzheimer’s, Parkinson’s, stroke and migraine (10). For instance, they utilized MR to reveal a causal relationship between cathepsins and neurological diseases, including Parkinson’s disease and ischemic stroke. These findings underscore the potential of MR in uncovering new avenues for therapeutic intervention. Zhao et al. used MR to investigate the causal effects of brain imaging phenotypes in migraine, providing insights into the neurophysiological changes underlying this common neurological disorder. A bidirectional MR study investigated the association between resting-state state functional activity (RSFA) and migraine. This study hypothesised that abnormalities in brain RSFA are causally associated with an increased risk of migraine. And suggest that certain brain networks, particularly those related to the visual cortex, may have a significant causal effect on migraine risk (11). A study published in 2022 shows that Parkinson’s disease and glioblastoma multiforme (GBM) may promote epigenetic ageing, providing new insights into the causal links between ageing and neurological disorders (12). Updated guidelines for the conduct of MR studies have been published, emphasizing the importance of accounting for horizontal pleiotropy, weak instrument bias and other potential violations of MR assumptions and these guidelines are critical to ensuring the validity of MR studies in neurological research (13). While MR methods have been extensively utilized in the study of cardiovascular and metabolic diseases (14, 15), it their application in the context of neurological diseases remains nascent. It is because that the application of MR in neurological research is also limited by the complexity of the brain and the multitude of factors that contribute to neurological diseases. The heterogeneity of these diseases and the influence of environmental factors add layers of complexity to establishing clear causal links (16). Moreover, there is a need for large-scale, high-quality genetic data specific to neurological disorders, which are not always readily available. The success of MR relies heavily on the availability of well-characterised genetic variants that are robustly associated with the exposure of interest (17). The implications of these MR studies for neurological research are profound. MR has the potential to transform our understanding of disease mechanisms and guide the development of more effective preventive and therapeutic strategies.

Therefore, it is of great importance to systematically sort out the application of MR methods in the study of neurological diseases in order to promote scientific progress in this field. This paper provides a comprehensive summary of the current status and emerging trends in the application of MR methods in the study of neurological diseases, utilizing BA.

BA, a methodological approach for examining the characteristics and trends within the literature, enables the evaluation and analysis of various dimensions such as quantity, quality, citation metrics, authorship, and institutional contributions. These methods allow for the analysis of trends over time, identifying emerging areas of research interest and shifts in the scientific paradigm (18). This methodology enables a rapid visualization of the current landscape of MR methods in neurological disease research, encompassing research hotspots, institutional collaborations, and prospective research directions. In this paper, we will conduct a systematic analysis of the characteristics of MR methods in this field, focusing on the volume of published literature, prevalent research topics, collaborative networks among research institutions, and the geographical distribution of research, utilizing the BA method. Furthermore, this study will assess the strengths and limitations of MR methods in neurological disease research and suggest potential directions for future research. Our aim is to provide neurological disease researchers with a comprehensive perspective on the application of MR methods and to facilitate interdisciplinary collaborations to accelerate scientific progress in this field.

## 2 Methods

### 2.1 Data source and literature search strategy

This study used data from the Science Citation Index Expanded (SCI-E) subset of the Web of Science Core Collection. With its extensive coverage of over 12,000 scientific journals and its widespread use by researchers, the Web of Science (WOS) is an ideal source for bibliometric data. Web of Science offers the most comprehensive and reliable BA among databases such as Scopus, Medline, and PubMed (19). SCI studies are typically characterised by a high level of academic rigor and influence within their respective fields and they are widely recognized as a benchmark for excellence within the academic community.

**Figure 1:**
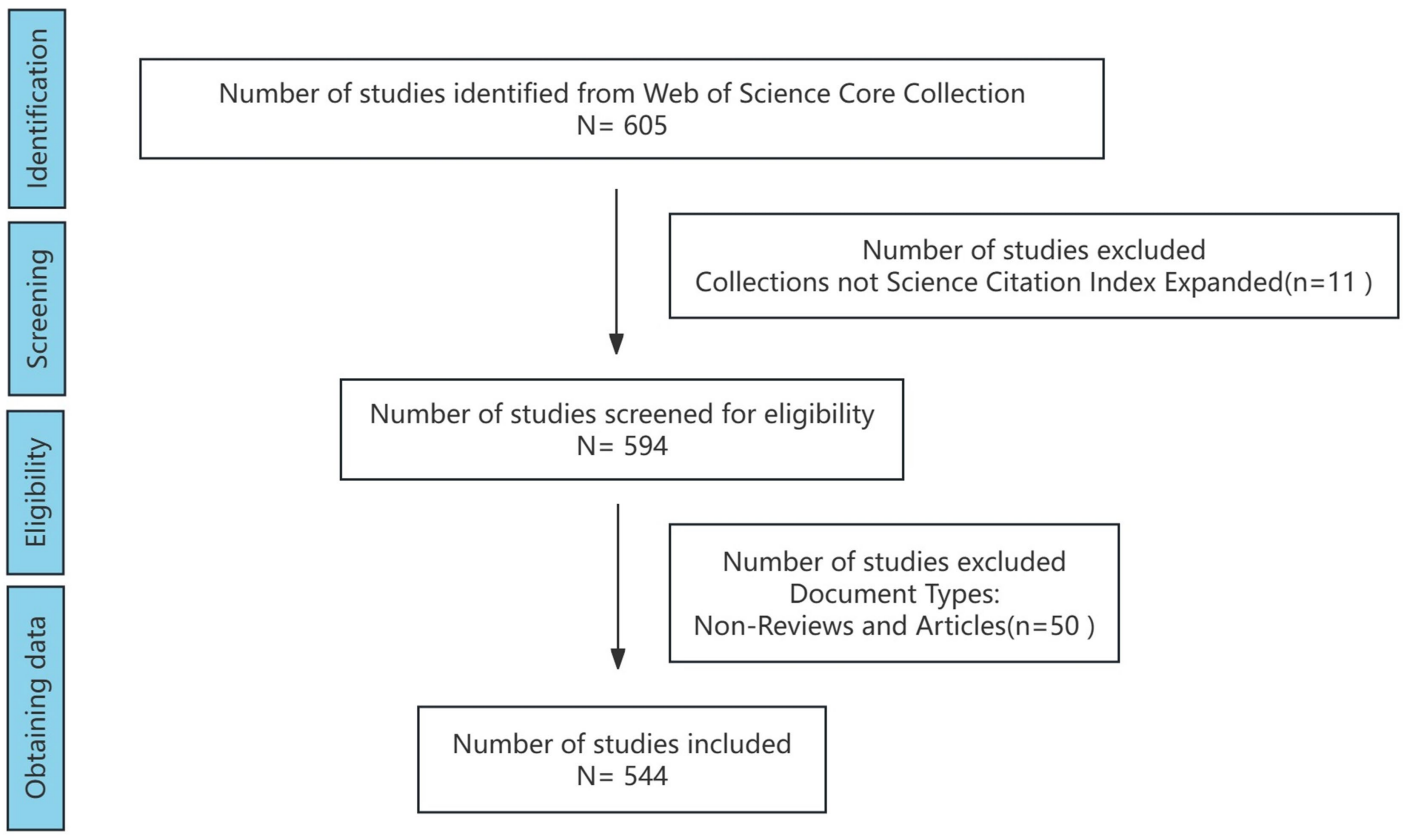
Literature retrieval and screening flow chart.

A comprehensive search was conducted for all articles indexed in the SCI published between January 1, 2014 and September 1, 2024, utilizing the search terms “neurological disorders” and “Mendelian randomization studies.” The data were analyzed on the day following their collation and exportation. Consequently, all data analyses presented in this article are current as of September 2024. The search parameters were intentionally broad, and various types of literature within the WOS database were included. The specific methodology employed for the literature search is detailed below: TS = ((“neurological diseases” OR “neurological disorder” OR “brain disorder” OR “brain injury” OR “central nervous system disease” OR “CNS disease” OR “central nervous system disorder” OR “CNS disorder” OR “stroke” OR “cerebrovascular diseases” OR “multiple sclerosis” OR “neurodegenerative diseases” OR “Alzheimer’s diseases” OR “Parkinson’s diseases” OR “traumatic brain injury” OR “acquired brain injury” OR “spinal cord injury” OR “cerebral palsy”) AND (“Mendelian Randomization Study”)). The initial search yielded 605 potentially relevant studies. Following a thorough screening process, we excluded 11 collections not indexed in the SCI-E, and the 594 documents were collated and placed into a folder labelled “Input”. Articles and reviews typically offer comprehensive background information, methodological approaches, findings, and discussions of the study, providing valuable data for analytical purposes. In contrast, non-research articles, such as newsletters, reviews, and case reports, may lack the requisite detail for in-depth bibliometric analyses. In particular, articles embody the latest research findings and original contributions within a specific field. Additionally, they offer fresh data sources for bibliometric analyses, which are instrumental in identifying emerging research trends and hotspots. Consequently, our selection for the thesis was confined to articles and review categories, excluding 50 items that did not meet the criteria for review or study types. This study adhered to the PRISMA guidelines (20) for reporting systematic reviews and meta-analyses. A comprehensive PRISMA flow diagram, presented in Figure 1, elucidates the study selection process. The literature search strategy was meticulously designed to ensure thoroughness, encompassing multiple databases and search terms. Studies were meticulously screened and selected based on predefined inclusion and exclusion criteria. The final cohort of 544 articles was compiled and stored in a folder designated “data”, where they underwent further analysis.

### 2.2 Statistical analysis

Upon the completion of literature collection, this study employed bibliometric analysis software to perform a thorough quantitative examination of the gathered literature. This analysis encompassed multiple dimensions, including fundamental information, developmental trajectories, and research hotspots. The elements analyzed comprised authors, countries, research institutions, publishing journals, references, and keywords. To uphold the accuracy and credibility of the data analysis, the processes of data extraction and analysis were meticulously delineated and executed independently. Furthermore, this study employed Bibliometrix software to visualize the research collaboration dynamics among countries and regions, as well as to analyze temporal trends and publication volumes in academic journals. And CiteSpace and VOSviewer were utilized to graphically represent the co-occurrence relationships among authors, keywords, journals, and research institutions. And CiteSpace and VOSviewer were utilized to graphically represent the co-occurrence relationships among authors, keywords, journals, and research institutions. The primary objective of these visualization tools is to elucidate the structure of research, identify prevailing patterns, and trace their propagation pathways. In the constructed network, the nodes symbolize research organizations, and the connecting lines between these nodes denote collaborative relationships. The primary objective of these visualization tools is to elucidate the structure of research, identify prevailing patterns, and trace their propagation pathways. In the constructed network, the nodes symbolize research organizations, and the connecting lines between these nodes denote collaborative relationships. The thickness of the connecting lines signifies the strength of these collaborative relationships, while the color gradient indicates the temporal onset of each collaboration. This intuitive methodology enables the study to illustrate the global research network and its evolutionary trends in the application of Mendelian randomization within the domain of neurological disorders.

## 3 Result

### 3.1 Analysis of annual publication and the publication trend

**Figure 2:**
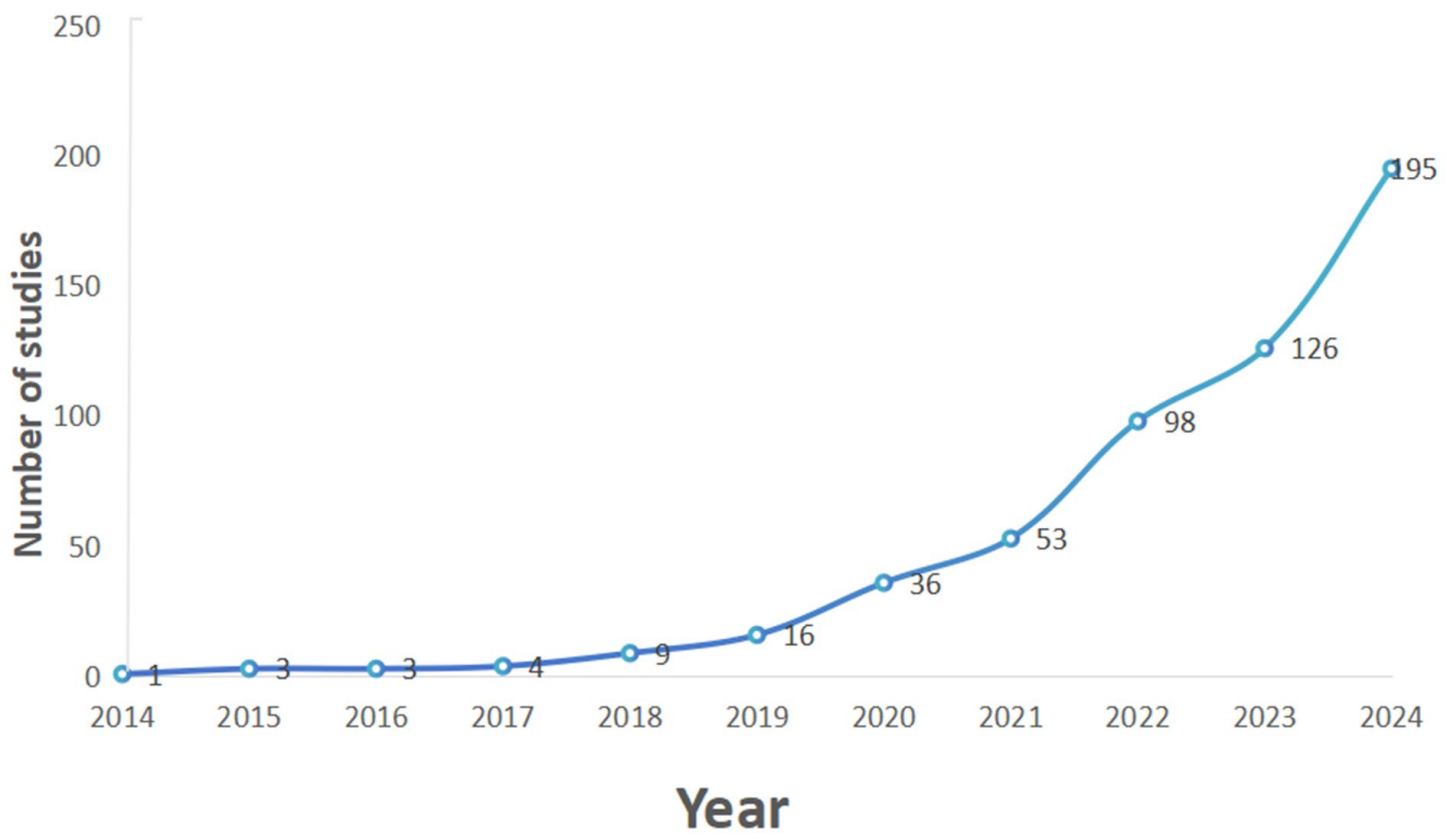
Annual publication of neurological diseases related mendelian randomization as of September 2024.

In this study, we conducted a systematic search of the WOCC databases to identify applications of MR imaging in neurological disease research. The publication trend in the literature indicates a notable increase in the number of pertinent studies since 2014, culminating in a peak in 2024 with a total of 195 published studies, as illustrated in Figure 2. This trend underscores the growing interest and application of Mendelian randomization as a causal inference methodology within the domain of neurological disorders. It is important to highlight that the preliminary data for 2024, despite being incomplete, indicate a sustained trend of growth, suggesting an increasingly significant role for the method in future research endeavors. These data not only offer a broad perspective on the application of MR methods but also establish a foundation for a more nuanced understanding of their value and potential in the context of neurological disease research.

### 3.2 Keyword analysis

**Figure 3:**
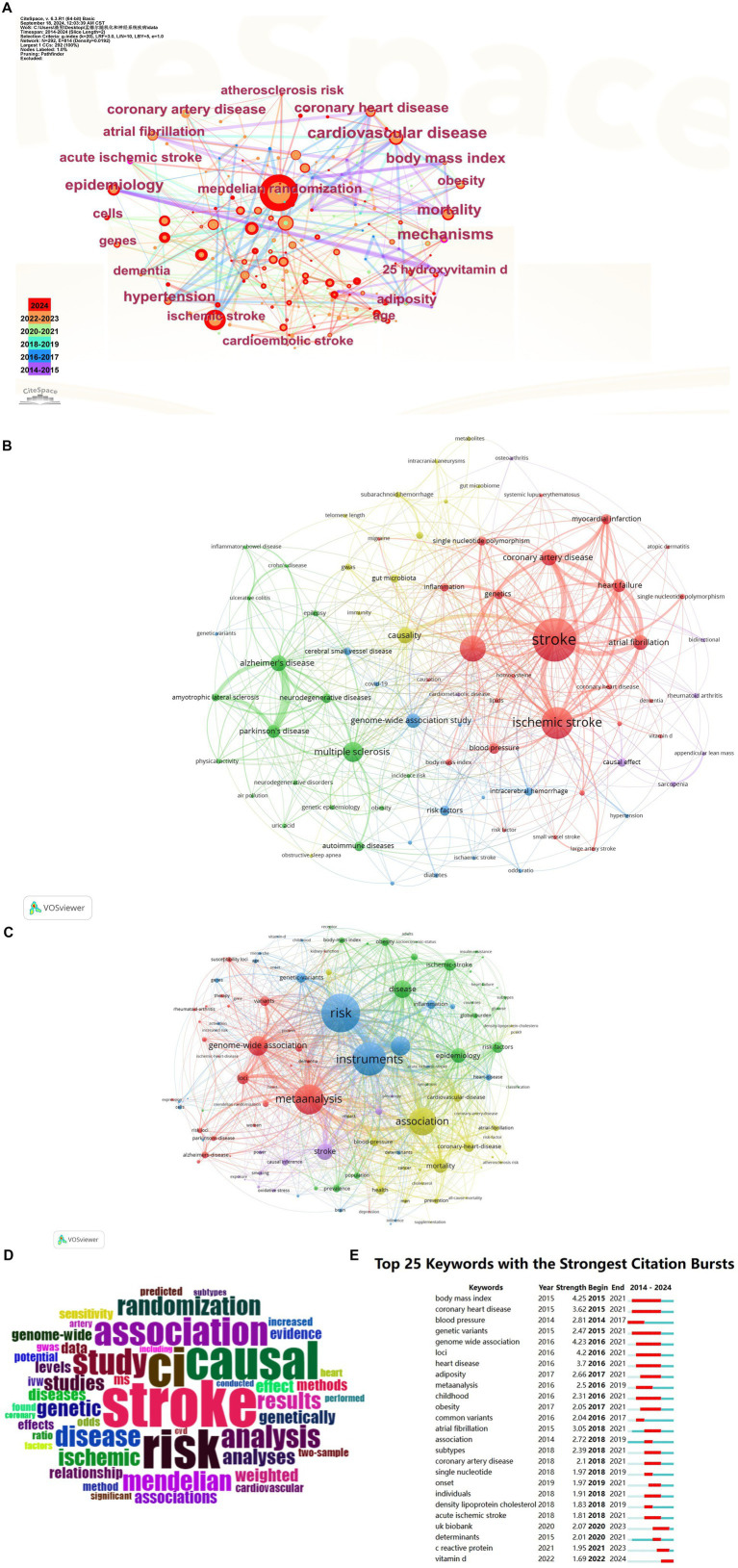
**(A)** Co-occurrence map of keywords. **(B)** Expansion of the author keyword co-occurrence. **(C)** Author’s keyword cooccurrence network. **(D)** Wordcloud of the abstracts. **(E)** Citation bursts for the top 25 keywords.

We analyzed keyword co-occurrences using CiteSpace and VOSviewer software. As illustrated in Figure 3A, our findings indicate that the terms “Mendelian randomization,” “ischemic stroke,” “cardiovascular disease,” and “genome-wide association” frequently appear in the literature, highlighting the predominant themes of contemporary research in this field. It is widely recognized that neurological diseases, particularly stroke, share numerous risk factors with cardiovascular diseases, including hypertension, diabetes mellitus, and hyperlipidemia. These factors not only elevate the risk of cardiovascular events but also contribute to the onset and progression of neurological disorders (21, 22).

The authors’ keywords predominantly centered on terms associated with “stroke” and “ischemic stroke,” underscoring the continued prominence of stroke as a fundamental research topic within the domain of neurological diseases, as illustrated in Figure 3B. In the expanded keyword analysis, alongside terms pertinent to neurological diseases, keywords directly aligned with the research objectives also emerged, including “risk,” “instruments,” “genome-wide association,” and “stroke,” as depicted in Figure 3C, suggesting that researchers are progressively employing systematic reviews and various statistical methodologies to enhance the reliability and generalizability of their investigations into stroke and other neurological disorders (23).

Additionally, the abstract indicates the term “stroke” occurring 1,750 times, followed by “ci” and “risk,” thereby underscoring the significance of stroke research and the emphasis on associated risk factors (24) (Figure 3D).

Keyword highlights not only encapsulate contemporary research topics but also suggest potential future research trajectories. Notably terms such as “body mass index,” “coronary heart disease,” and “blood pressure” have emerged prominently underscoring the significance of these variables in the investigation of the aetiology of neurological disorders. The prominence of these terms further emphasizes their critical role in understanding the underlying factors associated with neurological diseases (25, 26) as shown in Figure 3E. The pronounced increase in citations of “genetic variants” and “genome-wide association” underscores the growing scholarly focus on the role of genetic factors in neurological disorders (25, 27). The relationship between body mass index (BMI) and coronary heart disease has been a prominent area of investigation in the field of neurological disorders over the past decade. As time goes by the emergence of keywords such as “acute ischemic stroke” and “vitamin D” indicates that the relationship between specific diseases and nutrients in the context of neurological disorders is increasingly becoming a focal point of research (28).

The dynamic fluctuations of these keywords not only indicate a shift in research focus but also suggest potential future research directions, thereby offering new perspectives and strategies for the prevention and treatment of neurological diseases. Furthermore, genome-wide association studies (GWAS) have significantly contributed to the elucidation of the genetic determinants of complex diseases by analyzing extensive population DNA samples to identify disease-associated genetic variants, and enhances our understanding of the pathogenesis of complex human diseases (29). MR, an epidemiological method that uses genetic variation as an instrumental variable to assess causality, provides new perspectives on the impact of environmental exposures on disease (7).

In conclusion, investigations into neurological diseases extend beyond the examination of genetic and environmental risk factors; they increasingly incorporate a diverse array of advanced statistical methodologies and systematic evaluations. This convergence of research approaches serves as a robust framework for elucidating the intricate mechanisms underlying these diseases and for formulating novel preventive and therapeutic strategies.

### 3.3 Analysis of the cited articles

A comprehensive analysis of 299 publications, which collectively garnered 2,904 citations, includes three articles with 100 or more citations and ten publications with 50 or more citations. These findings underscore the significant potential of MR methods in elucidating causal relationships between genetic variants and health outcomes, as well as their broader applications in neurological research. Furthermore, these studies highlight the critical role of MR in evaluating antithrombotic therapy targets for stroke risk motif enrichment (30).

The independent causal effects of Mendelian randomization on various disease risk factors, including heart failure (HF), coronary artery disease (CAD), atrial fibrillation (AF), body mass index (BMI), and hypertension, were also investigated (31). And Rigor Enhancement Strategies for MR Research (32), significantly widens the boundaries of MR applications. At the same time, the study also focuses on raising the standard of MR reporting (33), provides guidance for peer review, clinical practice, and scientific interpretation. From the extension of query options to a new perspective based on expression spectra (34, 35). These papers illustrate the efficacy and innovation of MR methodologies in genetic association studies, particularly concerning the analysis of autoimmune processes within the central nervous system.

The extensively cited literature emphasizes the broad application and comprehensive advancement of MR in the investigation of neurological diseases. Furthermore, the ongoing refinement of analytical methods and reporting standards offers robust theoretical and technical support for the analysis of complex genetic etiologies, thereby advancing the field of precision medicine, detailed citation counts are presented in Table 1. The findings of the Malik R’s study (36) may be utilized to inform the development of prevention strategies. A deeper comprehension of the genetic underpinnings of stroke may facilitate the refinement of public health initiatives, thereby potentially reducing the incidence of stroke in high-risk populations. In light of the study’s multi-ancestry composition, its findings are applicable to a global population. This could facilitate the development of more inclusive health policies and clinical trials that take genetic diversity into account. This most cited article will continue to guide future research.

**Table 1:** Articles with the highest citations.

Rank	Author	Number of citations	Conclusion
1	Malik R (36)	181	It was found that loci associated with stroke risk were significantly enriched in antithrombotic drug targets.
2	Verbanck (55)	178	More than 48% of significant causal relationships in MR are detectable.
3	Gibran Hemani (52)	134	By integrating data with software, hypothesis-driven analyses can be applied more rigorously and millions of causal relationships can be evaluated more efficiently.
4	Mihir A Kamat (34)	74	There are now options for searching by genes, genomic regions, and phenotypes, as well as genetic variants.
5	Davies NM (56)	68	Interpretation of findings from Mendelian randomization studies in the context of other sources of evidence.
6	Shah S (31)	67	Mendelian randomization analysis indicates causal roles for multiple heart failure risk factors, revealing CAD-independent effects for atrial fibrillation, body mass index, and hypertension.
7	Skrivankova VW (33)	63	Reporting MR studies in accordance with the STROBE-MR guidelines would facilitate editors’ evaluations, peer reviewers’ evaluations, researchers’ interpretations and clinicians’ interpretations.
8	Bowden J (57)	60	Using the MBE in combination with other approaches in sensitivity analysis relaxes the instrumental variable assumptions.
9	Burgess Stephen (58)	60	A new version of these guidelines will be developed based on feedback from the community and advancements in the field.
10	Patsopoulos NA (59)	55	A study using purified human microglia indicated that they may play a role in the targeting of an autoimmune process to the central nervous system.

### 3.4 Analysis of countries and institutions

**Figure 4:**
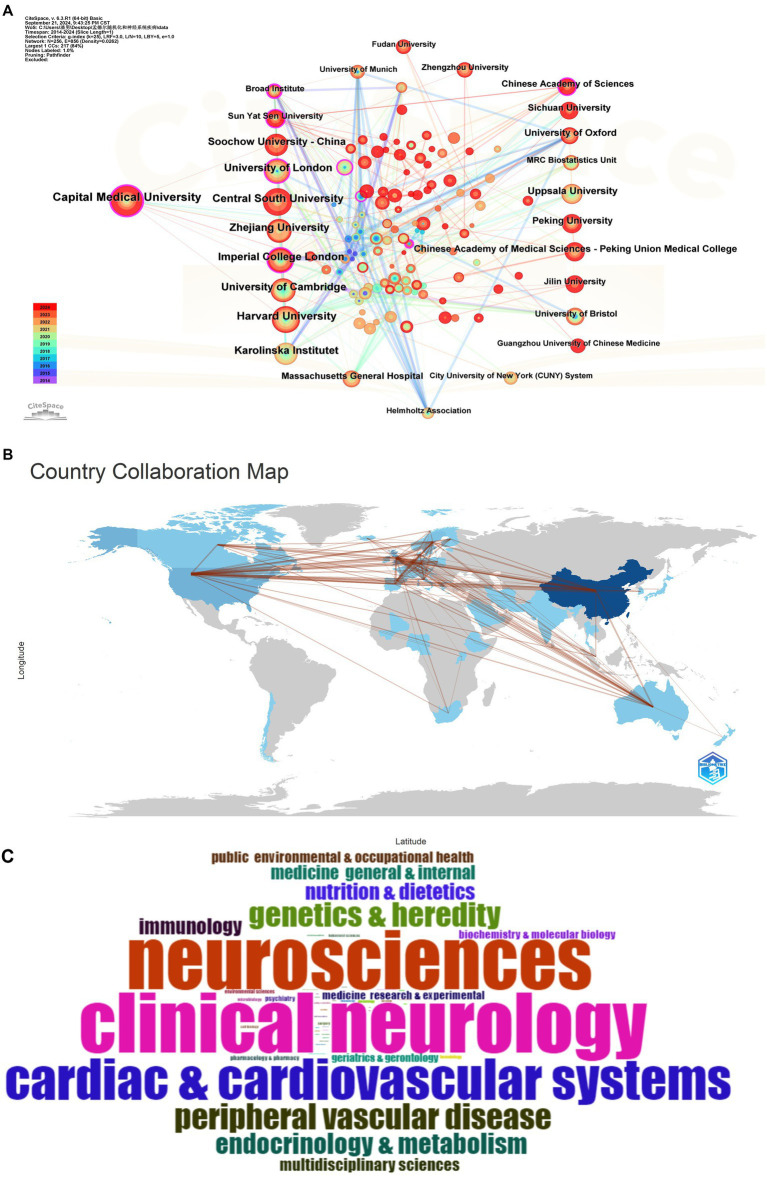
**(A)** Cooperation between countries. **(B)** Word cloud of the subject categories. **(C)** Inter-agency cooperation chart.

An examination of the national affiliations of the authors of the 544 extracted articles indicates that the majority of authors are based in China, followed by the United Kingdom, with the United States ranking third. Additionally, the analysis demonstrates that the most prevalent collaborations occur between authors from China and the United States, resulting in a total of 43 co-authored articles. This finding underscores the robust collaborative relationship between the two countries in the realm of scientific research, as depicted in Figure 4A. China’s collaboration with the United Kingdom is notably more substantial, encompassing a total of 29 partnerships. In contrast, the collaboration between the United Kingdom and Germany ranks third, with a total of 17 partnerships. This mode of transnational cooperation not only facilitates academic exchanges among various countries and regions but also enhances the globalization of scientific discovery.

At the level of specific disciplines, clinical neurology, neuroscience, and cardiovascular studies represent the three fields with the highest publication output, as illustrated in Figure 4B. This indicates that the MR methodology has extensive applicability across these domains, with researchers actively employing genetic tools to investigate the causal relationships of diseases.

At the institutional level, Capital Medical University leads with 50 published articles, followed by Central South University and Harvard University, which have published 36 and 35 articles, respectively, as illustrated in Figure 4C. In recent years, Capital Medical University has enhanced its collaborative efforts with various institutions, a trend that may correlate with an increase in its publication output. International collaborations facilitate the sharing of data and resources among countries, thereby accelerating scientific discovery and fostering advancements in the prevention and treatment of neurological disorders (Table 2).

**Table 2:** Articles published by the 10 most affiliated institutions.

Rank	Affiliated institutions	Centrality	Volume of publications
1	Capital Medical University	0.23	50
2	Central South University	0.02	36
3	Harvard University	0.08	35
4	Imperial College London	0.19	31
5	University of Cambridge	0.03	31
6	University of London	0.12	30
7	Zhejiang University	0.03	30
8	Karolinska Institutet	0.03	29
9	Soochow University – China	0.01	27
10	Uppsala University	0.04	22

### 3.5 Research co-occurrence of evolutionary trends

**Figure 5:**
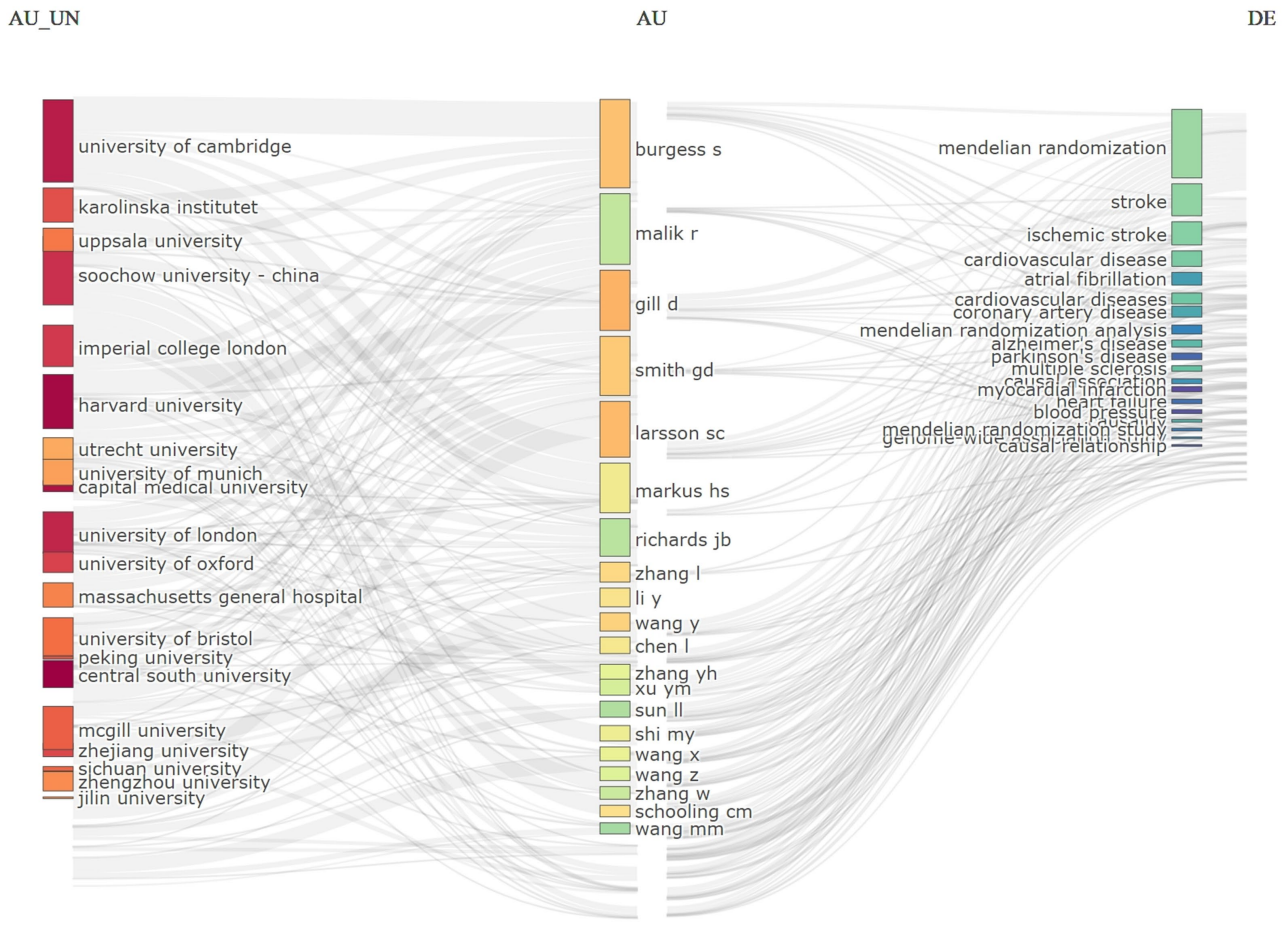
Network diagram of collaborations and thematic correlations in neurological disease research.

Figure 5 illustrates the global research networks and collaboration patterns within this domain. The figure highlights the prominence of leading academic institutions, including the University of Cambridge, Karolinska Institutet, and Soochow University in China, in the application of Mendelian randomization methods to neurological disease research. This prominence suggests that these institutions are instrumental in advancing the field. The contribution of researchers such as Markus HS (37, 38), Malik R (38), Gill D (39) in Mendelian randomization analysis is notable and their work emphasizes the role of genetic tools in exploring the coronary artery disease, cardiovascular diseases, Alzheimer’s disease, Parkinson’s disease and neurological disorders. The investigation of factors associated with cardiovascular and neurological diseases, including myocardial infarction, atrial fibrillation, ischemic stroke, and hypertension, has yielded novel genetic evidence that enhances our understanding of the underlying mechanisms of these diseases.

Furthermore, the intensity of the connecting lines in the figure serves as a visual representation of the degree of collaboration and the focal areas of the research. This interdisciplinary and inter-institutional collaborative network facilitates diverse avenues for exploring the intricate aetiology of neurological diseases and advances scientific progress from observational associations to causal inferences. This network map of collaborations and thematic associations in disease research illustrates the increasing significance of MR in the study of neurological diseases, while also emphasizing the critical role of international collaborations in addressing complex scientific challenges.

**Figure 6:**
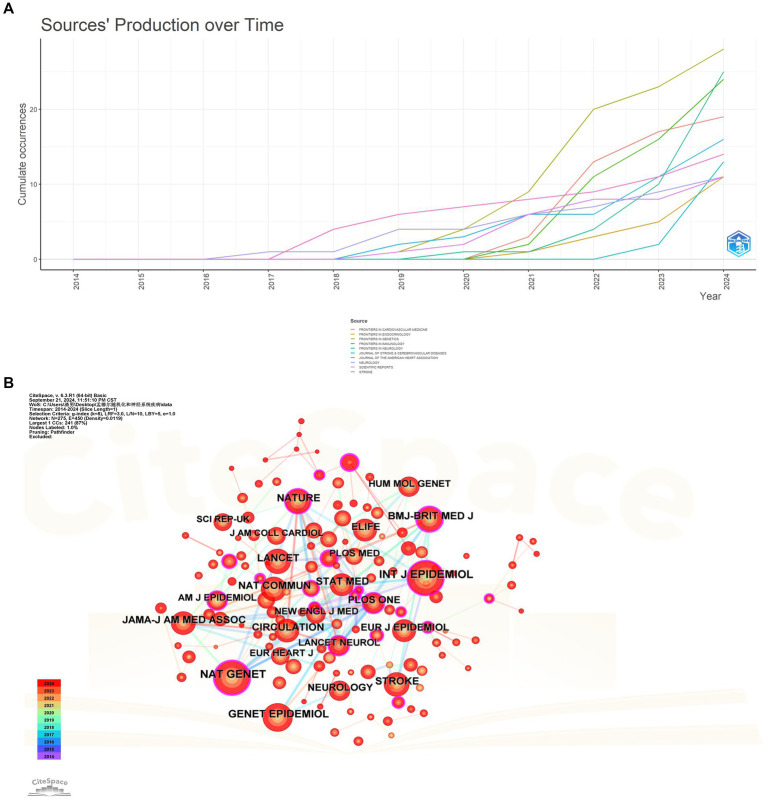
**(A)** Top 10 journals ranked by publications over time. **(B)** Chart of journal citations.

Among the various journals analyzed, FRONTIERS IN GENETICS published 28 relevant articles, FRONTIERS IN NEUROLOGY published 25, FRONTIERS IN IMMUNOLOGY published 24, ten other journals each published more than ten related papers, as illustrated in Table 3. Notably, the majority of the journals with the highest publication counts began disseminating relevant articles sequentially after 2018. Among these, FRONTIERS IN GENETICS, FRONTIERS IN IMMUNOLOGY, and FRONTIERS IN NEUROLOGY ave. demonstrated a consistent upward trend in the volume of relevant publications in recent years, as depicted in Figure 6A.

**Table 3:** Top ten journals in terms of publications.

Rank	Journal	IF	JCR	Articles
1	FRONTIERS IN GENETICS	2.8	Q2	28
2	FRONTIERS IN NEUROLOGY	2.7	Q2	25
3	FRONTIERS IN IMMUNOLOGY	5.7	Q1	24
4	FRONTIERS IN CARDIOVASCULAR MEDICINE	2.8	Q2	19
5	JOURNAL OF THE AMERICAN HEART ASSOCIATION	5	Q1	16
6	STROKE	7.8	Q1	14
7	JOURNAL OF STROKE & CEREBROVASCULAR DISEASES	6	Q1	13
8	FRONTIERS IN ENDOCRINOLOGY	3.9	Q2	11
9	NEUROLOGY	7.7	Q1	11
10	SCIENTIFIC REPORTS	2.8	Q1	11

IF, impact faction; JCR, journals citation reports.

In particular, while FRONTIERS IN IMMUNOLOGY primarily concentrates on immunology, its notable impact factor and substantial volume of publications in this field indicate a growing interest in the investigation of immune factors in neurological disorders.

The co-occurrence mapping of journals elucidates the co-citation network within this research domain, while the analysis of the top ten journals by publication volume underscores the academic journals that contribute the most significant research. The journal co-occurrence map illustrates that publications such as JOURNAL OF THE AMERICAN MEDICAL ASSOCIATION (JAMA), NEW ENGLAND JOURNAL OF MEDICINE, and NATURE GENETICS exhibit a high co-citation frequency concerning MR and its applications to neurological diseases. This elevated co-citation rate underscores the academic influence and authority of these journals within the field. Furthermore, a total of 31 journals have received more than one hundred citations among those in which the related literature was published, as depicted in Figure 6B.

## 4 Discussion

This study presents significant findings derived from an econometric analysis of the literature concerning the application of MR in neurological disorders. These results contribute to a comprehensive understanding of the current status and emerging trends in this research domain.

An examination of annual publication metrics reveals that pertinent articles first emerged in 2014, with a marked increase in publication volume beginning in 2021, indicating a consistent year-on-year growth in the body of literature in recent years. This trend clearly indicates that the application of MR in the study of neurological disorders is progressively garnering widespread attention, with increasing research interest. This growth is likely closely associated with the rapid advancements in genetics and epidemiological techniques in recent years, which have rendered the application of MR methods in neurological disease research more feasible and effective (40, 41).

Co-occurrence network analysis identified several significant themes. The terms “ischemic stroke,” “cardiovascular disease,” and “genome-wide association” emerged as prevalent concepts that accurately represent the primary focus of contemporary research (42, 43). The terms “body mass index” and “coronary heart disease” were prominently featured among the most frequently cited keywords, indicating a significant association between neurological disorders and cardiovascular disease risk factors (26). For instance, research conducted by Wang, ML et al. (44) and Vargas-Soria, M et al. (45) have established that risk factors associated with cardiovascular disease, including hypertension, diabetes mellitus, and hyperlipidemia, not only elevate the likelihood of cardiovascular events but also markedly intensify the onset and progression of neurological disorders (44, 46). Furthermore, the authors’ keywords predominantly centered on “stroke” and “ischemic stroke,” thereby underscoring the significance of stroke within the context of neurological disease research (47). Concurrently, the ongoing innovation and integration of research methodologies—including the extensive application of systematic evaluations and statistical techniques, alongside the comprehensive advancement of genome-wide association studies-have significantly contributed to elucidating the intricate mechanisms underlying neurological disorders and to the formulation of effective preventive and therapeutic strategies, as corroborated by the findings of de Klein et al. (48). This is consistent with existing research highlighting the genetic basis of these conditions and underlines the potential of MR to elucidate causal relationships in these areas.

In the context of research collaboration and evolutionary trends, there has been a notable increase in cooperation and exchange among various research institutions, including the University of Cambridge, Karolinska Institutet, Uppsala University, and other distinguished entities within the field, thereby establishing a comprehensive collaborative network. Initial investigations concentrated on specific neurological disorders; however, over time, the scope of research has progressively broadened to encompass a wider array of neurological conditions, with a deeper exploration of the application of MR in these disorders (49, 50). Future research trends are anticipated to emphasize multidisciplinary cross-fertilization, integrating insights from diverse fields such as genetics, neuroscience, and epidemiology. This approach aims to elucidate the relationship between MR and neurological diseases in a more comprehensive manner. Such interdisciplinary collaboration is expected to transcend the limitations of traditional research methodologies, thereby introducing novel perspectives and methodologies to the investigation of neurological diseases (51).

The analysis of the cited literature reveals that a total of 299 articles have been referenced, accumulating 2,904 citations. Notably, three of these articles have received over 100 citations, while ten articles have been cited more than 50 times. This pattern of citation underscores the considerable potential of MR methods in elucidating causal relationships between genetic variants and health outcomes, as well as their extensive application and significant influence within the domain of neurological research. For instance, the research conducted by Malik et al. offers valuable insights into the role of MR in assessing the enrichment of stroke risk loci associated with antithrombotic therapy targets, thereby contributing novel perspectives for the prevention and treatment of stroke (36). Similarly, the study by Shah et al. investigates the independent causal effects of MR on various disease risk factors, thereby establishing a critical foundation for a comprehensive understanding of the pathogenesis of neurological disorders (31). The study conducted by Gibran Hemani et al. focused on enhancing the rigor of MR research, thereby ensuring the reliability and credibility of its findings (52). The results of this research have established a robust foundation for the continued advancement of the field and have facilitated the ongoing refinement and application of MR methodologies in the investigation of neurological diseases.

In the context of country and institutional analysis, this study revealed that the majority of the articles were authored by researchers from China, followed by those from the United Kingdom and the United States. Collaborations between China and the United States were found to be the most prevalent, with partnerships between China and the United Kingdom also demonstrating considerable significance. Additionally, collaborations between the United Kingdom and Germany ranked third in frequency. This pattern of transnational collaboration not only facilitates academic exchanges across various countries and regions but also contributes to the acceleration of the globalization of scientific discovery. In the realm of subject areas, clinical neurology, neuroscience, and the cardiovascular system emerge as the three domains with the highest volume of published articles, suggesting a significant application of MR method within these fields. Capital Medical University (CMU), Central South University (CSU), and Harvard University (HU) lead the rankings in terms of publication output. Notably, CMU has enhanced its collaborative efforts with other institutions in recent years, a development that may be closely associated with its increased publication rate through international cooperation. It highlights the importance of international collaboration, in line with global scientific trends that emphasize the value of collaborative research in advancing the understanding and treatment of neurological disorders.

In terms of journal analysis, FRONTIERS IN GENETICS, FRONTIERS IN NEUROLOGY, and FRONTIERS IN IMMUNOLOGY have published a significant volume of literature on related topics. Notably, the majority of the top ten journals, ranked by the number of published articles, began to release related publications sequentially after 2018. Furthermore, NAT GENET, INT J EPIDEMIOL, and GENET EPIDEMIOL are among the most frequently cited journals, with a total of 31 journals exceeding one hundred citations. These journals serve as a crucial platform for disseminating the findings of MR studies in the field of neurological diseases, thereby facilitating academic discourse and the exchange of knowledge.

The increasing accessibility of data from genome-wide association studies (GWAS) has improved the identification of genetic variants associated with neurological disorders. In Mendelian randomization (MR) studies, these genetic variants serve as instrumental variables, allowing researchers to explore the causal connections between genetic influences and disease outcomes. The trends observed reflect a move towards interdisciplinary collaboration, with researchers from genetics, epidemiology and neurology working together, which is essential for a comprehensive understanding of complex neurological diseases. Neurological disorders are highly heterogeneous, which may contribute to the wide range of genetic variants and risk factors studied using MR methods. This heterogeneity also highlights the need for personalised medicine approaches, which MR can help to inform.

Consequently, these findings indicate that MR methods have significant potential for applications in the study of neurological disorders. This is supported by previous studies that have used MR to identify causal relationships between genetic variants and health outcomes, thereby reinforcing the validity and utility of the method. The growing collaboration among various countries and institutions, coupled with ongoing advancements in research methodologies and technologies, has facilitated novel approaches and strategies for the investigation and treatment of neurological disorders. Future research must prioritize a more comprehensive interdisciplinary cross-fertilization among multiple fields, rigorously investigate the intricate mechanisms underlying neurological diseases, and enhance both the breadth and depth of international collaboration. These efforts are essential for facilitating significant advancements in scientific progress within this domain. Additionally, for certain contentious findings—particularly regarding the associations between specific risk factors and neurological diseases—further studies are necessary to validate and elucidate these relationships, thereby ensuring the accuracy and reliability of the results (12, 53). MR provides a unique lens through which to view the complex interplay between genetic factors, modifiable risk factors and neurological disease. By circumventing some of the traditional challenges associated with observational studies, MR offers a promising avenue for uncovering new insights into the aetiology of neurological diseases and identifying potential therapeutic targets. A growing recognition in the scientific community of the importance of multidisciplinary approaches to complex problems such as neurological disorders. The results of our study contribute to current scientific understanding by highlighting the growing interest and application of MR in neurological research. This may influence future research directions and resource allocation within the field.

## 5 Advantages and limitations

The MR approach offers substantial advantages in the investigation of neurological disorders, as it employs genetic variation as an instrumental variable, thereby simulating the conditions of randomized controlled trials within observational study frameworks. This methodology effectively mitigates the influence of confounding variables and reverse causality, thereby facilitating novel insights into potential causal pathways in complex neurological disorders. MR provides a means of establishing causality by using genetic variants as proxies for environmental exposures, thereby reducing the impact of confounding that is often present in observational studies (7). MR has been instrumental in identifying novel biomarkers for neurological diseases. A recent study integrated machine learning with MR to identify PALMD as a prognostic biomarker for non-specific orbital inflammation, highlighting the role of genetic predisposition in disease incidence (54). Nonetheless, the application of MR method is subject to certain limitations, including the potential for pleiotropy associated with genetic variants and the risk of insufficient statistical power arising from the limited capacity of these genetic variants to account for exposure factors. And MR studies are susceptible to weak instrumental bias, which can occur when the genetic variants used as instruments have a small effect on exposure (7). The applicability of MR findings to diverse populations may be limited due to differences in genetic architecture between populations.

The strength of this study resides in its comprehensive examination of the current status and emerging trends regarding the application of MR methods in the investigation of neurological disorders, achieved through a systematic BA. By leveraging the extensive data resources of the Web of Science Core Collection database, in conjunction with sophisticated BA software and visualization tools, this research elucidates the characteristics of research hotspots, collaborative networks, and the geographic distribution of MR methods within this domain from a macroscopic perspective. Furthermore, this study employs quantitative analysis and graphical representation to elucidate the strengths and limitations of MR methods in the investigation of neurological disorders, thereby offering potential guidance for future research trajectories.

However, the limitations of this study must be taken into account. First, BA relied on the selection of specific databases, which may introduce biases in the analysis. For example, this could lead to a biased analysis if certain regions or types of studies are under-represented in this database. Reliance on a single database may also miss relevant literature published in other repositories or in languages other than English. This is typically associated with the selected time point. For instance, our study uses a specific cut-off date (1 September 2024), which could influence the number of citations an article receives. Articles published closer to the cut-off date may not have had enough time to accumulate citations, potentially underestimating their impact. Additionally, visualization analysis may be inadequate in conveying the complex relationships and structures inherent in research, even though it offers an intuitive representation. The complex interrelationships between researchers, institutions and concepts could be oversimplified. Moreover, the limitations associated with the chosen literature and keywords may have influenced the outcomes of this study. MR findings may not be generalisable to different populations due to differences in genetic architecture. This may limit the applicability of the results, particularly in populations with different genetic backgrounds. Therefore, future research should aim to validate and expand upon them.

## 6 Conclusion

In this study, BA is used to demonstrate the growing trend of MR in neurological disease research. A high number of related publications is reported every year, as is the focus on specific diseases and risk factors, as well as better collaboration among institutions. A major focus should be placed on multidisciplinary collaborations, deepening our knowledge of disease mechanisms, and improving international collaborations in the future. However, the application of MR method has certain limitations, including the potential pleiotropy of genetic variants and the risk of insufficient statistical power due to their limited ability to account for exposure factors. It is necessary to develop a dedicated genetic database for neurological diseases. Research in this field has advanced positively, providing new directions and hope for neurological disease treatment.

## Data Availability

Publicly available datasets were analyzed in this study. This data can be found at: web of science.
